# Serum bilirubin value predicts hospital admission in carbon monoxide-poisoned patients. Active player or simple bystander?

**DOI:** 10.6061/clinics/2015(09)06

**Published:** 2015-09

**Authors:** Gianfranco Cervellin, Ivan Comelli, Ruggero Buonocore, Alessandra Picanza, Gianni Rastelli, Giuseppe Lippi

**Affiliations:** I Emergency Department, Academic Hospital of Parma; II Laboratory of Clinical Chemistry and Haematology, Academic Hospital of Parma, Italy; III Emergency Department, Hospital of Vaio, Fidenza, Italy

**Keywords:** Lactate, Carboxyhemoglobin, Bilirubin, Carbon Monoxide, Poisoning

## Abstract

**OBJECTIVES:**

Although carbon monoxide poisoning is a major medical emergency, the armamentarium of recognized prognostic biomarkers displays unsatisfactory diagnostic performance for predicting cumulative endpoints.

**METHODS:**

We performed a retrospective and observational study to identify all patients admitted for carbon monoxide poisoning during a 2-year period. Complete demographical and clinical information, along with the laboratory data regarding arterial carboxyhemoglobin, hemoglobin, blood lactate and total serum bilirubin, was retrieved.

**RESULTS:**

The study population consisted of 38 poisoned patients (23 females and 15 males; mean age 39±21 years). Compared with discharged subjects, hospitalized patients displayed significantly higher values for blood lactate and total serum bilirubin, whereas arterial carboxyhemoglobin and hemoglobin did not differ. In a univariate analysis, hospitalization was significantly associated with blood lactate and total serum bilirubin, but not with age, sex, hemoglobin or carboxyhemoglobin. The diagnostic performance obtained after combining the blood lactate and total serum bilirubin results (area under the curve, 0.90; 95% CI, 0.81-0.99; *p<*0.001) was better than that obtained for either parameter alone.

**CONCLUSION:**

Although it remains unclear whether total serum bilirubin acts as an active player or a bystander, we conclude that the systematic assessment of bilirubin may, alongside lactate levels, provide useful information for clinical decision making regarding carbon monoxide poisoning.

## INTRODUCTION

Although carbon monoxide (CO) poisoning is a major medical emergency, resulting in more than 50,000 emergency department (ED) visits each year in the United States 1, the pathophysiology of this condition is still incompletely understood. It is widely assumed that CO can cause hypoxia by directly binding to hemoglobin molecules with an affinity up to 200 times greater than that of oxygen for hemoglobin, thus leading to the generation of carboxyhemoglobin (COHb) and a left shift in oxyhemoglobin dissociation, which ultimately decrease oxygen delivery to peripheral tissues 1. Nevertheless, other, more complex mechanisms have been recently postulated, such as the generation of enhanced oxidative stress due to a direct increase in cytosolic heme levels 2 or the direct binding of CO to tissue heme proteins and cytochrome C oxidase 3, which severely compromises cellular respiration and increases the endogenous generation of reactive oxygen species (ROS) 4. This pernicious biochemical cascade not only leads to cell injury, inflammation and metabolic acidosis 1 but also triggers a defensive response from the organism, which is mainly mediated by the activation of several antioxidant and anti-inflammatory mechanisms 5,6.

Notably, CO can also be generated by physiological mechanisms during toxic exposure, and more specifically by heme metabolism, as initially demonstrated by Sjöstrand more than sixty years ago 7,8. A class of enzymes, namely, the heme oxygenases (HOs), share the function of metabolizing the heme molecule. This process is associated with the generation of equimolar amounts of CO and biliverdin, the latter of which is then promptly converted into bilirubin by a specific reductase 9. Interestingly, HO is present in two genetically distinct isoforms, or HO-1 and HO-2. The latter isoenzyme is constitutively expressed, whereas the former HO-1 isoenzyme belongs to the family of heat-shock proteins, and its synthesis can be induced by a wide spectrum of chemical and physical stressor agents, including hypoxia and CO 10,11. Bilirubin, one of the products generated by heme metabolism, is currently regarded as a natural antioxidant and a potent scavenger of ROS 12,13. Recent evidence also suggests that serum bilirubin levels inversely correlate with cardiovascular diseases and mortality 14,15.

Because COHb levels are poor predictors of hospitalization and outcome in CO-poisoned patients 16,17, increased focus is currently being placed on the identification of additional indices of the severity and outcome of CO exposure. In particular, a limited amount of evidence of the predictive value of cardio-specific troponins 18, lactate 19,20, copeptin 21 and protein S-100B 22 in CO-poisoned patients is accumulating.

Due to the intriguing relationship that exists between bilirubin and CO metabolism, the goal of this study was to assess whether total serum bilirubin levels, which are considered as the expression of the anti-inflammatory response of the organism to poisoning, has a role in predicting the short-term outcome (i.e., the need for hospitalization) of CO-poisoned patients.

## MATERIALS AND METHODS

In this retrospective and observational study, all patients referred to the ED of the Academic Hospital of Parma due to CO poisoning from 2012-2013 were identified in the hospital database. According to local practice, arterial COHb, hemoglobin and blood lactate are routinely measured in all patients with suspected CO poisoning in the ED itself using the blood gas analyzer Radiometer ABL800 FLEX (Radiometer Medical ApS, Bronshoj, Denmark), whereas total serum bilirubin is assessed in the central laboratory using a Beckman Coulter AU5800 instrument and the azobilirubin assay (Beckman Coulter Inc., Brea, CA, USA). For the purposes of this investigation, CO poisoning was diagnosed only in the presence of an arterial COHb concentration greater than 2% in non-smokers and greater than 9% in smokers, in association with a history suggestive of recent CO exposure along with clinical features of CO poisoning. At our institution, the hospitalization of a CO-poisoned subject is determined by the emergency physician in charge of the patient, according to clinical judgment. All cases were of domestic origin, i.e., due to problems with the heating system in the patient's house.

The normality of the distribution of values was verified using the Shapiro-Wilk test. The data were found to be non-normally distributed and were thus ultimately reported as the median and interquartile range (IQR). The significance of differences between discharged and hospitalized patients was assessed with the Wilcoxon-Mann-Whitney test. Univariate analysis (Spearman's correlation) was then performed to define potential associations between clinical outcome data and demographical or laboratory data. The statistical analysis was performed using Analyse-it (Analyse-it Software Ltd, Leeds, UK). The study was performed in accordance with the Declaration of Helsinki and under the terms of relevant local legislation.

## RESULTS

Complete information could be found for 38 CO-poisoned patients (23 females and 15 males; mean age 39±21 years) from the retrospective analysis of 2012-2013 admissions. Among these patients, 10 (26%) were hospitalized for insufficient recovery after acute treatment and/or clinical signs of organ dysfunction (principally for heart injury and/or cardiac dysfunction). No deaths occurred in the 2 weeks following initial admission to the ED. The main findings of this study are summarized in table 1. Compared with the discharged subjects, the patients in the hospitalized group were older and displayed significantly higher values for both blood lactate and total serum bilirubin, whereas the hemoglobin and arterial COHb values did not differ significantly between the groups. The range of total serum bilirubin values in the whole population of CO-poisoned patients (i.e., 3.4-21 μmol/L) largely overlapped with the normal reference range of the local laboratory (3-20 μmol/L).

In the univariate analysis, hospitalization was found to be significantly associated with blood lactate and total serum bilirubin, but not with age, sex, hemoglobin or COHb. Additionally, strong positive correlations were found between blood lactate and CO (r=0.417; *p=*0.009) and between hemoglobin and total serum bilirubin (r=0.430, *p=*0.026).

**Figure 1:**
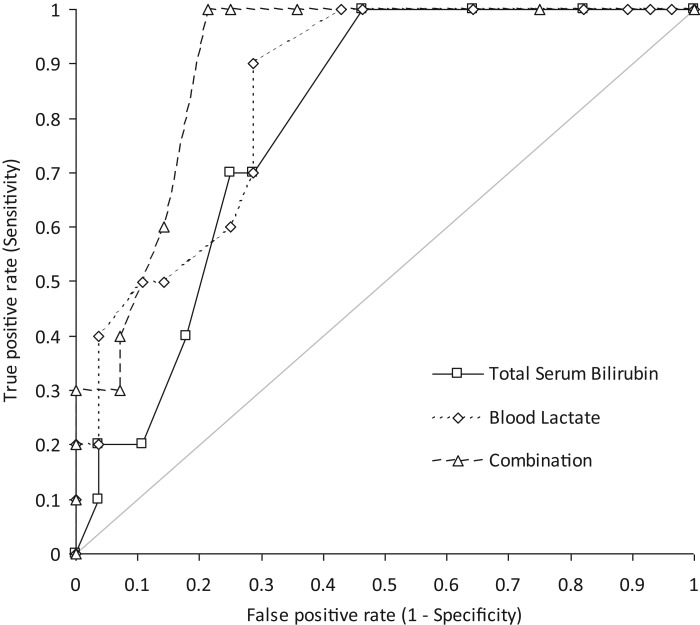
Diagnostic performance (receiver operating characteristic curve) of the admission values of total serum bilirubin, blood lactate and their combination for predicting hospitalization in carbon monoxide-poisoned patients.

The area under the curve (AUC) for predicting hospitalization was 0.85 (95% CI, 0.72-0.97; *p<*0.001) for blood lactate and 0.79 (95% CI, 0.65-0.93; *p<*0.001) for serum bilirubin. At a threshold of >1.20 mmol/L blood lactate, the sensitivity and specificity for predicting hospitalization were 1.00 (95% CI, 0.69-1.00) and 0.57 (95% CI, 0.37-0.76), respectively. At a threshold of total serum bilirubin >6.8 μmol/L, the sensitivity and specificity for predicting hospitalization were 1.00 (95% CI, 0.69-1.00) and 0.54 (95% CI, 0.34-0.72), respectively. Interestingly, the diagnostic performance obtained after combining the blood lactate and total serum bilirubin results (AUC, 0.90; 95% CI, 0.81-0.99; *p<*0.001) was better than that obtained for either parameter alone (Figure 1). The sensitivity and specificity of this combination (both parameters with cut-off values obtained from receiver operating characteristic (ROC) curves) were 1.00 (95% CI, 0.69-1.00) and 0.79 (95% CI, 0.59-0.92), respectively.

## DISCUSSION

The results of this study are the first to show that the total serum bilirubin level at patient admission in the ED is a significant predictor of hospitalization among CO-poisoned patients and that its combination with the blood lactate value predicts hospital admission with high accuracy (AUC, 0.89). It is also noteworthy that the total serum bilirubin concentration per se did not influence the decision regarding hospital admission or discharge because the values were virtually identical to those of the normal reference range.

There is an intriguing and mutual relationship between CO, lactate and bilirubin, which may provide a reasonable explanation for these findings. As previously reported, HO is a key enzyme in bilirubin metabolism because it catalyzes the conversion of heme into bilirubin. More specifically, HO participates in the rate-limiting first step of the oxidative degradation of Fe-protoporphyrin-IX into biliverdin, which is subsequently converted into bilirubin by an NAD(P)H-dependent reductase. The process of biliverdin generation, which requires three molecules of molecular oxygen (O_2_) and one molecule of NADPH, generates one molecule of ferrous iron (i.e., Fe^2+^) along with one molecule of CO (9). Recent evidence suggests that HO-1 may confer protection against oxidative stress by enhancing the generation of biliverdin, bilirubin and iron, given that biliverdin and bilirubin are powerful endogenous antioxidant compounds 14,15. In contrast, the iron liberated from heme by HO stimulates the production of ferritin, which is a well-known cytoprotective molecule 9.

Considering this evidence along with the results of our study, it is conceivable that a greater degree of tissue hypoxia in CO-intoxicated patients, as reflected by higher blood lactate values, may effectively stimulate the synthesis of HO-1, which would subsequently enhance the in vivo generation of CO and bilirubin. This hypothesis is partially confirmed by the strong positive correlation between blood lactate and CO observed in this study. A similar pathway could be suggested for CO because a higher concentration of COHb may also effectively induce overexpression of HO-1, thereby enhancing bilirubin synthesis. From this perspective, increased levels of total serum bilirubin should be considered as a bystander, rather than as an effective player. Conversely, it is expected that the higher the conversion of heme to biliverdin (and then to bilirubin) is, the greater the generation of endogenous CO will be. Thus, in patients with a high basal hemoglobin value, increased generation of bilirubin may occur under stress conditions, such as CO-related hypoxia. This concept mirrored by the significant correlation between hemoglobin and total serum bilirubin observed in our patients. This intriguing relationship may ultimately be effective in augmenting and maintaining an increased level of endogenous CO, for which bilirubin levels may also be considered as an active player, rather than as a simple bystander. This scenario is consistent with evidence that CO exerts its effects as both a signaling molecule 13 and a highly hypoxic agent 23.

COHb levels are poor predictors of hospitalization and outcome in CO-poisoned patients 16,17, and increased emphasis is currently being placed on the identification of additional indices of the severity and outcome of CO exposure. Among the most useful biomarkers, evidence of the predictive value of cardio-specific troponins 18, lactate 19,20, copeptin 21 and protein S-100B 22 in CO-poisoned patients is accumulating. Among these biomarkers, as previously reported, blood lactate seems to be the only significant predictor of hospital admission 20. Nevertheless, these biomarkers only reflect organ-specific derangements (i.e., brain or heart injury); hence, they display an overall unsatisfactory diagnostic performance in terms of predicting cumulative endpoints such as hospitalization or death 1.

Although it remains unclear whether total serum bilirubin acts as an active player or a simple bystander, it seems reasonable to conclude that the systematic assessment of this simple and inexpensive parameter may provide, alongside lactate levels, useful information for clinical decision making regarding CO poisoning.

## Figures and Tables

**Table 1:** Values (median and interquartile range) for 38 patients with acute carbon monoxide poisoning, stratified according to hospital admission, and univariate analysis (Spearman's correlation) for the prediction of hospitalization in carbon monoxide-poisoned patients.

	No. hospitalizations (28)	Hospitalizations (10)	*p*-value	Spearman's correlation
Age (years)	35 (25-45)	45 (37-25)	0.035	r=0.297; *p=*0.070
Gender (Females/Males)	18/10	5/5	0.342	r=0.123; *p=*0.441
Hemoglobin (g/L)	135 (123-145)	124 (120-134)	0.274	r=-0.120; *p=*0.548
Carboxyhemoglobin (%)	15.0 (8.1-21.4)	18.8 (12.4-30.0)	0.091	r=0.221; *p=*0.181
Blood lactate (mmol/L)	1.0 (1.1-1.5)	1.8 (1.4-2.0)	<0.001	r=0.534; *p<*0.001
Total serum bilirubin (µmol/L)	6.8 (5.1-9.3)	10.3 (9.0-13.0)	0.005	r=0.417; *p=*0.009

## References

[1] Weaver LK (2009). Clinical practice. Carbon monoxide poisoning. N Engl J Med.

[2] Cronje FJ, Carraway MS, Freiberger JJ, Suliman HB, Piantadosi CA (2004). Carbon monoxide actuates O(2)-limited heme degradation in the rat brain. Free Radic Biol Med.

[3] Alonso JR, Cardellach F, López S, Casademont J, Miró O (2003). Carbon monoxide specifically inhibits cytochrome c oxidase of human mitochondrial respiratory chain. Pharmacol Toxicol..

[4] Piantadosi CA, Penney D G (1996). Toxicity of carbon monoxide: hemoglobin vs. histotoxic mechanisms. Carbon monoxide.

[5] Mannaioni PF, Vannacci A, Masini E (2006). Carbon monoxide: the bad and the good side of the coin, from neuronal death to anti-inflammatory activity. Inflamm Res.

[6] Chin BY, Jiang G, Wegiel B, Wang HJ, Macdonald T, Zhang XC (2007). Hypoxia-inducible factor 1 alpha stabilization by carbon monoxide results in cytoprotective preconditioning. Proc Natl Acad Sci U S A.

[7] Sjöstrand T (1949). Endogenous production of carbon monoxide in man under normal and pathophysiological conditions. Scandinavian Journal of Clinical and Laboratory Investigation.

[8] Sjöstrand T (1952). The formation of carbon monoxide by the decomposition of hemoglobin in vivo. Acta Physiol Scand..

[8] Ryter SW, Alam J, Choi AMK (2006). Heme oxygenase-1/carbon monoxide: From basic science to therapeutic applications. Physioll Rev..

[9] Chung HT, Ryter SW, Kim HP (2013). Heme oxygenase-1 as a novel metabolic player. Oxid Med Cell Longev..

[10] Yang YC, Huang YT, Hsieh CW, Yang PM, Wung BS (2014). Carbon Monoxide Induces Heme Oxygenase-1 to Modulate STAT3 Activation in Endothelial Cells via S-Glutathionylation. PLoS One.

[11] Stocker R, Ames BN (1987). Potential role of conjugated bilirubin and copper in the metabolism of lipid peroxides in bile. Proc Natl Acad Sci U S A.

[12] Breimer LH, Mikhailidis DP (2010). Could carbon monoxide and bilirubin be friends as well as foes of the body. Scand J Clin Lab Invest..

[13] Breimer LH, Spyropolous KA, Winder AF, Mikhailidis DP, Hamilton G (1994). Is bilirubin protective against coronary artery disease. Clin Chem.

[14] Franchini M, Targher G, Lippi G (2010). Serum bilirubin levels and cardiovascular disease risk: a Janus Bifrons. Adv Clin Chem..

[15] Hampson NB, Hauff NM (2014 Jun). Carboxyhemoglobin levels in carbon monoxide poisoning: do they correlate with the clinical picture. Am J Emerg Med..

[16] Hampson NB, Piantadosi CA, Thom SR, Weaver LK (2012). Practice Recommendations in the Diagnosis, Management, and Prevention of Carbon Monoxide Poisoning. Am J Respir Crit Care Med..

[17] Lippi G, Rastelli G, Meschi T, Borghi L, Cervellin G (2012). Pathophysiology, clinics, diagnosis and treatment of heart involvement in carbon monoxide poisoning. Clin Biochem..

[18] Moon JM, Shin MH, Chun BJ (2011). The value of initial lactate in patients with carbon monoxide intoxication: in the emergency department. Hum Exp Toxicol..

[19] Cervellin G, Comelli I, Rastelli G, Picanza A, Lippi G (2014). Initial blood lactate correlates with carboxyhemoglobin and clinical severity in carbon monoxide poisoned patients. Clin Biochem..

[20] Pang L, Wang HL, Wang ZH, Wu Y, Dong N, Xu DH (2014). Plasma copeptin as a predictor of intoxication severity and delayed neurological sequelae in acute carbon monoxide poisoning. Peptides.

[21] Cakir Z, Aslan S, Umudum Z, Acemoglu H, Akoz A, Turkyilmaz S (2010). S-100&bgr; and neuron-specific enolase levels in carbon monoxide&ndash;related brain injury. Am J Emerg Med.

[22] Guzman JA (2012). Carbon monoxide poisoning. Crit Care Clin.

